# Evolutionary and anthropological perspectives on the sella turcica: from vertebrate origins to neurosurgical relevance

**DOI:** 10.1007/s40618-025-02698-y

**Published:** 2025-09-04

**Authors:** M. Vaccarezza, S. Taurone, M. Palmieri, F. M. Galassi, L. Cofone, M. Artico, V. Papa

**Affiliations:** 1 Curtin Medical School, Curtin University, Perth, WA Australia; 2 Curtin Medical Research Institute, Curtin University, Perth, WA Australia; 3 Department of Movement, Human and Health Sciences, Division of Health Sciences, University of Rome “Foro Italico”, Rome, Italy; 4 Neurosurgery Division, Human Neurosciences Department, “Sapienza” University of Rome, Rome, Italy; 5 Department of Anthropology, Faculty of Biology and Environmental Protection, University of Lodz, Łódz, Poland; 6 Department of Sense Organs, “Sapienza” University of Rome, Rome, Italy; 7 Department of Economics, Law, Cybersecurity, and Sports Sciences, University of Naples “Parthenope”, Naples, Italy; 8 School of Science, Engineering and Health, University of Naples “Parthenope”, Naples, Italy

**Keywords:** Sella turcica, Evolutionary anatomy, Pituitary gland, Craniometric analysis, Paleopathology, Hominin evolution, Endocrine system development

## Abstract

The sella turcica, a saddle-shaped depression of the sphenoid bone, serves as a critical anatomical structure housing the pituitary gland and holds significant evolutionary, clinical, and anthropological importance. This review traces the evolutionary origins of the sella turcica from early vertebrates through mammalian and primate evolution, emphasizing its role in the stabilization and protection of neuroendocrine functions. Morphological stability of the sella turcica across hominin evolution highlights strong selective pressures on cranial base anatomy, despite broader craniofacial diversification. Anthropologically, the sella turcica provides a durable landmark for craniometric analyses, forensic reconstructions, and paleoanthropological investigations, revealing patterns of sex-based dimorphism, population variation, and disease prevalence. Developmental anomalies such as empty sella syndrome and pituitary hypoplasia illustrate the evolutionary trade-offs between increased encephalization and cranial vulnerability. Integrating historical, paleopathological, and clinical perspectives, this article underscores the sella turcica’s significance as a nexus of evolutionary innovation, structural resilience, and biological fragility.

## Introduction

The sella turcica, a saddle-shaped depression in the body of the sphenoid bone, constitutes a crucial element of the human cranial base. It houses and protects the pituitary gland, the “master gland” orchestrating endocrine functions essential to growth, metabolism, reproduction, and systemic homeostasis [1]. Although extensively studied for its clinical implications in endocrinology and neurosurgery, the sella turcica also commands attention from evolutionary and anthropological perspectives. Its emergence and morphological refinement across vertebrate phylogeny mirror the centralization of neuroendocrine systems and the increasing complexity of vertebrate physiology [2]. Moreover, as a stable craniometric landmark, the sella turcica provides critical insights into craniofacial development, pathological deviations, and evolutionary adaptation [3]. Recent historical reviews, such as those by Cucu et al., emphasize how anatomical understanding of the pituitary gland—and by extension the sella turcica—evolved from ancient misconceptions about nasal mucus drainage to the sophisticated endocrinological models of modern neurosurgery [4, 5]. Through comparative anatomy, fossil analysis, and anthropological studies, the sella turcica emerges as both a functional necessity and an evolutionary hallmark.

## Evolutionary origins of the Sella turcica

The evolutionary roots of the sella turcica can be traced to the earliest gnathostomes (jawed vertebrates) of the Devonian period (approximately 419–359 million years ago). Fossilized skulls of placoderms and early cartilaginous fishes exhibit cartilaginous recesses accommodating nascent neurovascular structures [6]. Although ossified sellar structures were absent, these depressions hint at an early strategy to shield sensitive endocrine tissues. As vertebrates transitioned to terrestrial environments, particularly during the rise of amphibians and reptiles, cranial depressions homologous to the human sella turcica appeared more prominently [7]. The stabilization of the sellar region was driven by the increasing centralization of neuroendocrine regulation. As growth, stress responses, and reproduction demanded precise control, evolutionary pressures favored the development of a stable, centrally located bony enclosure [8]. Thus, the sella turcica represents an anatomical response to the escalating complexity of vertebrate systemic regulation, aligning with the broader evolutionary trajectory of endocranial specialization.

### Development through mammalian evolution

Mammalian evolution brought dramatic modifications to the cranial base, resulting in a well-defined and consistent sella turcica across taxa. In early synapsids, cranial differentiation began with elaboration of the sphenoid bone and development of foramina for neurovascular structures [9]. Fossil evidence from early eutherian mammals, such as *Eomaiascansoria* (circa 125 million years ago), reveals a distinct endocranial depression, suggestive of the progressive anatomical integration of the hypothalamic-pituitary axis [9]. The evolution of the sella turcica was closely tied to the demands of mammalian biology, including prolonged gestation, thermoregulation, and maternal lactation. Basicranial flexion, a key feature in primates, shifted the pituitary gland closer to the geometric center of the cranial vault, enhancing its protection and integration with cerebral structures [10]. Studies in non-human primates show that basicranial flexure improved mechanical stability while optimizing hormonal communication pathways [11]. The human sella turcica thus reflects a long evolutionary journey balancing cranial expansion, neurological sophistication, and systemic regulation. Recent research suggests that relaxed natural selection may increase phenotypic variability in modern human populations. Although this mechanism has been primarily studied in relation to somatic traits like body mass [12], future research may explore its potential implications for craniofacial structures such as the sella turcica.

## Historical evolution of anatomical Understanding

Historically, understanding of the pituitary gland and its anatomical housing lagged behind its functional importance. Ancient theories, dating back to Galen, considered the gland a drainage site for brain waste [4, 5]. This misconception, rooted in Hippocratic thought, persisted until the Renaissance, when Vesalius first depicted the gland as a separate anatomical entity [4, 5]. Subsequent anatomical studies by Schneider, Lower, and Willis progressively corrected earlier views, suggesting circulatory rather than excretory functions [4, 5]. By the 18th century, anatomists such as Santorini and von Haller recognized the anterior and posterior divisions of the pituitary [4, 5]. The 19th century witnessed the beginnings of clinical correlations between pituitary pathology and systemic diseases, with Verga, Marie, and Minkowski linking sellar tumors to conditions such as acromegaly [4, 5]. These insights set the stage for the emergence of experimental pituitary surgery in the late 19th and early 20th centuries, culminating in the pioneering work of Harvey Cushing [4, 5].

## Anthropological significance

Anthropologically, the sella turcica serves as a remarkably resilient anatomical marker, playing a crucial role in craniometric analysis, forensic reconstruction, and population-specific studies. Its deep-seated location at the base of the skull, surrounded by dense cranial structures, enables it to withstand considerable postmortem degradation, making it one of the most consistently preserved features in archaeological and forensic contexts [13]. This durability allows for reliable measurements even in fragmentary remains, thus providing a foundational reference point for assessing cranial morphology, reconstructing facial structures, and estimating biological profiles in both ancient and modern populations. Comparative paleoanthropological studies of fossil hominins, notably *Homo erectus* and *Homo neanderthalensis*, reveal a relative morphological consistency in the dimensions and positioning of the sella turcica, despite substantial differences in overall cranial vault size, facial prognathism, and brain volume [11].

This morphological conservation across diverse evolutionary lineages supports the hypothesis that the functional imperative of safeguarding the pituitary gland—a central regulatory hub for endocrine function—imposed strong stabilizing selection pressures throughout hominin evolution. In other words, while other cranial features underwent adaptive modifications in response to environmental, dietary, and locomotor challenges, the anatomical integrity of the sellar region was maintained as a non-negotiable evolutionary priority. Modern anthropological research further illustrates that the sella turcica exhibits subtle but consistent sex-based dimorphism, with males generally displaying larger and deeper sellar depressions than females [14]. This sexual variation likely correlates with broader trends in endocranial volume and cranial base dimensions, and it offers a valuable metric for sex estimation in osteological analysis. In addition to sex-based differences, geographic and ancestral variations in sella turcica morphology have been documented. Population-specific studies demonstrate slight but measurable differences in sellar size, shape, and contour among African, Asian, and European groups, reflecting both adaptive responses to environmental pressures and the effects of neutral genetic drift [15]. These variations, while relatively minor, contribute to broader patterns of craniofacial diversity and provide additional parameters for bioarchaeological reconstructions and evolutionary modelling. Beyond normal anatomical variation, pathological alterations of the sella turcica, such as sellar enlargement secondary to pituitary adenomas or erosive changes from chronic endocrinopathies, can be detected in skeletal remains. These abnormalities offer critical windows into the health profiles, disease burdens, and life histories of past populations [16]. The identification of such pathologies not only enriches our understanding of ancient disease prevalence and the biological consequences of endocrine dysregulation but also reinforces the anthropological value of the sella turcica as a dynamic record of both normal and pathological human development. As bioarchaeological methodologies continue to advance, integrating imaging technologies and 3D morphometric analyses, the study of the sella turcica promises to yield even finer-grained insights into the complex interplay between biology, environment, and culture in shaping the human experience.

## Pathological variations and evolutionary Trade-offs

Developmental and congenital anomalies affecting the sella turcica—including conditions such as empty sella syndrome, pituitary hypoplasia, and sellar dysplasia—underscore the intricate evolutionary trade-offs that accompanied the anatomical and functional advancements of the human cranial base. As hominins evolved larger and increasingly complex brains, profound mechanical and developmental pressures were exerted on the central structures of the cranial base, particularly the region of the sella turcica [8]. The architectural refinements necessary to accommodate both expanded encephalization and delicate neuroendocrine pathways introduced zones of vulnerability where small perturbations in developmental processes could lead to significant structural and functional disruptions. Empty sella syndrome, characterized by the herniation of cerebrospinal fluid into the sellar space with consequent flattening or atrophy of the pituitary gland, offers a poignant example of the anatomical compromises shaped by evolutionary cranial base remodeling [17]. This heterogenous condition likely reflects a delicate balance between the demands of supporting a larger, flexed brain and maintaining the integrity of intracranial pressure gradients across developmental stages. Similarly, pituitary hypoplasia, involving the congenital underdevelopment of the pituitary gland, illustrates the fine-tuned orchestration required between neurocranial expansion, vascular supply, and endocrine organogenesis. Deviations in these tightly regulated processes reveal the cost of evolutionary innovations: systems designed for optimized performance under ideal conditions became susceptible to malfunction when perturbed. Paleopathological analyses further substantiate the evolutionary depth of these vulnerabilities. Skeletal remains from ancient populations have exhibited evidence of sellar anomalies, such as expanded or eroded sellae consistent with pituitary adenomas or other endocrine disorders, indicating that dysregulation of the hypothalamic-pituitary axis is not a uniquely modern pathology but has deep evolutionary roots [16–19]. These findings suggest that the anatomical and functional refinements that enabled the sophisticated neuroendocrine regulation essential to human adaptability also carried an intrinsic risk of pathological disruption—a testament to the evolutionary principle that biological complexity often comes at the cost of increased fragility.

## Conclusion

Far from being a minor anatomical feature, the sella turcica embodies a profound evolutionary, functional, and anthropological narrative. From its nascent cartilaginous precursors in ancient fishes to its refined form in modern humans, the sella turcica reflects the intertwined evolution of cranial architecture and systemic regulation. Historical shifts in anatomical understanding mirror broader advancements in biomedical knowledge. As interdisciplinary research continues to bridge evolutionary biology, anthropology, and clinical medicine, the study of the sella turcica promises to yield even deeper insights into the biological compromises and innovations that have shaped the human lineage.
